# Hyperfibrinolysis during intra-aortic balloon pump support: a case report on targeted tranexamic acid therapy

**DOI:** 10.3389/fcvm.2025.1746269

**Published:** 2026-01-02

**Authors:** Shanshan Dong, Xinshuang Chen, Qi Peng, Jun Yang, Qimei Wei

**Affiliations:** 1 Department of Clinical Laboratory, Wuhan Asia Heart Hospital, Wuhan, China; 2 Department of Cardiac Critical Care Medicine, Wuhan Asia Heart Hospital, Wuhan, China

**Keywords:** case report, heart failure, intra-aortic balloon pump, secondary hyperfibrinolysis, tranexamic acid

## Abstract

Coagulation disturbances in patients with end-stage heart failure receiving intra-aortic balloon pump (IABP) support present significant management challenges. We describe a 49-year-old male with dilated cardiomyopathy awaiting transplantation who developed secondary hyperfibrinolysis following IABP-associated infection and hemodynamic instability. The patient exhibited pronounced D-dimer elevation (peak: 55.84 μg/mL) and persistent oozing at the puncture site. At the onset of hyperfibrinolysis, laboratory tests demonstrated a markedly increased plasmin-α2-plasmin inhibitor complex (PIC: 26.56 μg/mL) and a mildly elevated thrombin-antithrombin III complex (TAT: 7.89 ng/mL), accompanied by a rise in platelet count (351 × 10⁹/L, up from 318 × 10⁹/L previously) and a decrease in fibrinogen (4.85 g/L, down from 7.98 g/L). Targeted intravenous tranexamic acid (TXA) therapy effectively controlled bleeding and corrected fibrinolysis, without inducing thrombotic complications, thereby allowing successful bridging to heart transplantation. This case underscores the importance of considering secondary hyperfibrinolysis in IABP-supported patients with infection or hemodynamic instability.

## Introduction

Patients with end-stage dilated cardiomyopathy frequently require mechanical circulatory support to maintain hemodynamic stability while awaiting heart transplantation. The intra-aortic balloon pump (IABP) remains a cornerstone of bridging therapy in this population due to its minimally invasive nature and procedural feasibility (1). However, IABP use is accompanied by a spectrum of complications, including limb ischemia (incidence: 1.3%–7.5%), catheter-related infection (2%–10%), bleeding (3%–33%), and thromboembolic events, such as stroke (approximately 3.8%) (2–5). Among these, coagulopathy is a particularly challenging complication.

While previous research has primarily addressed common complications such as disseminated intravascular coagulation and heparin-induced thrombocytopenia (6), secondary hyperfibrinolysis remains underrecognized in both clinical characterization and management. Secondary hyperfibrinolysis is defined by excessive plasminogen activation, leading to a heightened risk of abnormal bleeding and, paradoxically, thrombosis. Its pathogenesis may involve mechanical injury, inflammatory cytokine release, and endothelial dysfunction (7). In patients with advanced heart failure, the interplay of continuous endothelial trauma from the IABP catheter, superimposed infections (e.g., sepsis), and tissue hypoperfusion related to low cardiac output may collectively precipitate dysregulation of the fibrinolytic system (4, 6). Nevertheless, reports of IABP-associated secondary hyperfibrinolysis are exceedingly rare, and evidence-based management strategies are lacking.

Here, we present a case of secondary hyperfibrinolysis in a patient with end-stage dilated cardiomyopathy during IABP support, triggered by catheter-related infection and progressive cardiac dysfunction. The diagnosis was established through serial monitoring of fibrinolytic biomarkers, and targeted tranexamic acid (TXA) therapy was successfully employed to control bleeding and facilitate heart transplantation. This report aims to highlight the risk and diagnostic considerations of secondary hyperfibrinolysis during IABP support, discuss the therapeutic role and safety profile of TXA in this context, and examine the complexities of coagulation management in patients with multiple interacting risk factors. Through this case, we seek to provide practical insights for clinical management.

## Case presentation

A 49-year-old man was admitted on February 18, 2022, with a 6-year history of intermittent chest tightness and dyspnea, initially exertional and relieved by rest. Six years prior, he was diagnosed with dilated cardiomyopathy and managed pharmacologically. Two years before admission, he underwent cardiac resynchronization therapy with defibrillator (CRT-D) implantation (hereafter referred to as ICD) for worsening cardiac function, resulting in symptomatic improvement. Eight months prior to this admission, the patient was hospitalized at another institution to optimize cardiac function and await heart transplantation. For the purpose of pursuing further treatment, the patient was transferred to our hospital for care. At admission, diagnoses included dilated cardiomyopathy and severe mitral regurgitation. Past medical history was notable for 10 years of hemorrhoids. The patient denied any other significant comorbidities.

Upon examination, the patient was alert and oriented. Cardiac assessment revealed marked cardiomegaly with displacement of the left and inferior cardiac borders. The heart rate was regular at 62 bpm. Pulmonary auscultation demonstrated coarse breath sounds bilaterally, without rales. Laboratory evaluation showed normal hepatic and renal function, and inflammatory markers (WBC, procalcitonin, hs-CRP) were within reference ranges. Coagulation studies indicated a prothrombin time of 15.6 s (reference: 9.6–12.3 s) and APTT of 36.3 s (reference: 24.6–35.4 s). A D-dimer of 0.166 μg/mL (reference: 0–0.5 μg/mL), fibrinogen of 3.61 g/L (reference: 2–4 g/L), and platelet count of 224 × 10⁹/L (reference: 125–350 × 10⁹/L) were also observed. N-terminal pro-B-type natriuretic peptide was elevated at 5,083 pg/mL. Echocardiography demonstrated significant biatrial and left ventricular enlargement, diffuse left ventricular hypokinesia, a severely reduced left ventricular ejection fraction of 25%, and moderate mitral regurgitation.

Standard heart failure therapy was initiated, including inotropes, diuretics, and anticoagulation. Due to further cardiac deterioration, an IABP was placed on day 5 with standard heparin anticoagulation (unfractionated heparin 1,000 U/h), resulting in hemodynamic stabilization. On day 13 post-IABP, the patient developed fever (39.5 °C) and local pain with discharge at the insertion site. Catheter-related infection was diagnosed; wound cultures grew *Klebsiella pneumoniae*, but blood cultures remained negative. Targeted ceftazidime therapy was started. During treatment, the patient experienced malignant ventricular arrhythmia, successfully terminated by ICD defibrillation and resuscitation.

On IABP day 33, the patient developed a high fever (40 °C), accompanied by bleeding at the insertion site and an elevated hs-CRP (153.02 mg/L). Suspecting recurrent infection, IABP was removed and antibiotic therapy escalated to piperacillin-tazobactam plus vancomycin. Cardiac function deteriorated following IABP removal, necessitating reinsertion the next day. Serial D-dimer monitoring revealed a progressive increase (from 0.701 μg/mL to 9.745 μg/mL, then 55.839 μg/mL) despite a platelet count that was elevated compared to previous readings (increasing from 318 × 10⁹/L to 351 × 10⁹/L). CT angiography excluded aortic dissection and pulmonary embolism. Further investigation revealed elevated plasmin-α2-plasmin inhibitor complex (PIC: 26.56 μg/mL; reference: 0–0.8 μg/mL) and thrombin-antithrombin complex (TAT: 7.89 ng/mL; reference: 0–4 ng/mL), while fibrinogen showed a decreasing trend (from 7.98 g/L to 4.85 g/L), further supporting the diagnosis of secondary hyperfibrinolysis.

**Figure 1:**
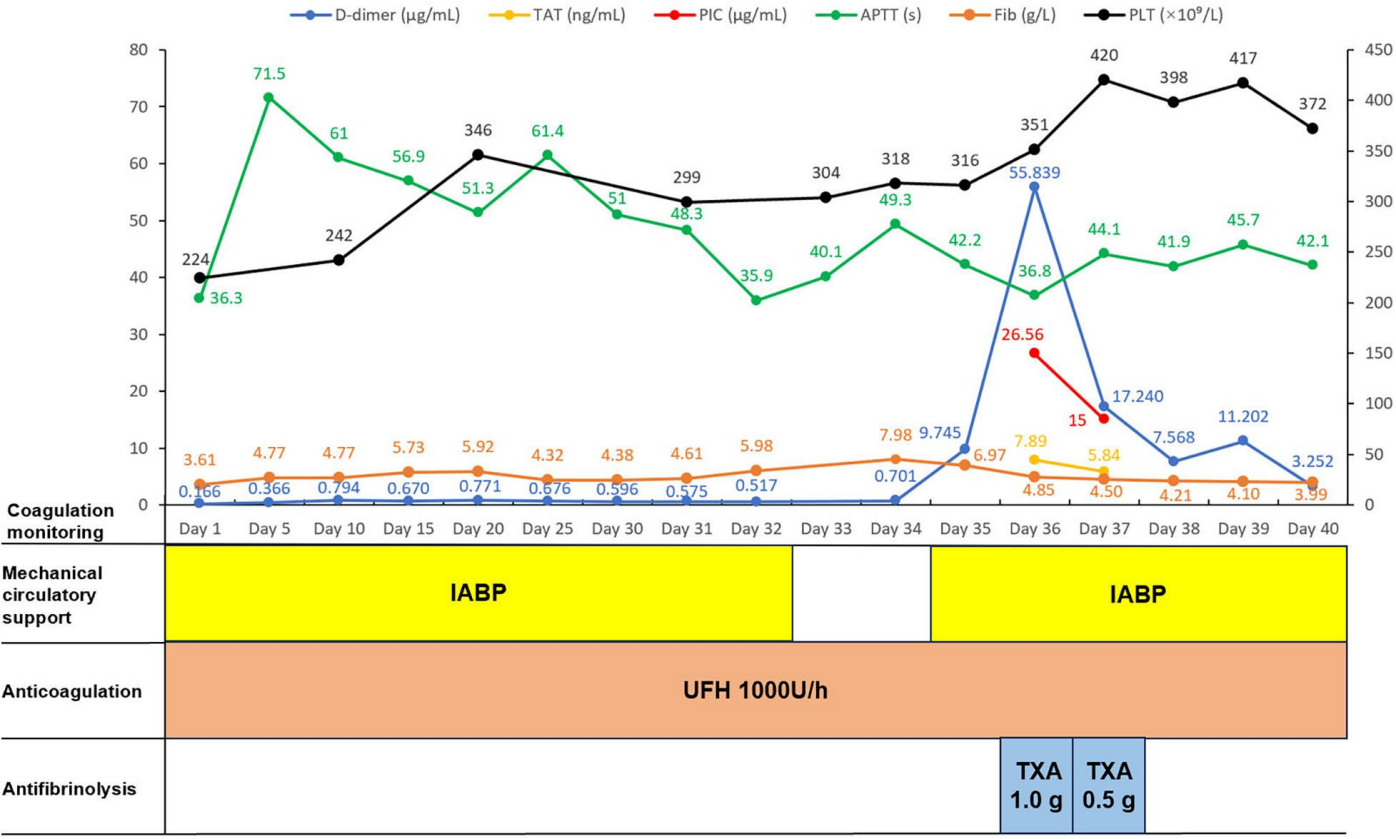
Coagulation monitoring and corresponding therapeutic interventions. TAT, thrombin-antithrombin complex; PIC, plasmin-α2-plasmin inhibitor complex; APTT, activated partial thromboplastin time; Fib, fibrinogen; PLT, platelet; IABP, intra-aortic balloon pump; UFH, unfractionated heparin; TXA, tranexamic acid.

TXA 1.0 g was administered, followed by a reduction in D-dimer and PIC levels (to 17.24 μg/mL and 15.0 μg/mL, respectively) the next day. An additional TXA dose (0.5 g) was given, with continued improvement in fibrinolytic parameters (Figure 1). The patient's condition stabilized with effective anti-infective and antifibrinolytic therapy. After multidisciplinary assessment, he underwent successful orthotopic heart transplantation under cardiopulmonary bypass on April 4, 2022. The postoperative course was uneventful, with no major bleeding or thrombotic complications, and the patient was discharged on postoperative day 23.

## Discussion

We present a case of IABP-induced secondary hyperfibrinolysis in a patient with end-stage dilated cardiomyopathy, successfully treated with intravenous TXA. TXA administration achieved hemostasis and correction of fibrinolytic abnormalities without precipitating thrombotic events, facilitating subsequent heart transplantation. This case underscores the complexity of coagulation disturbances in critically ill cardiac patients and the importance of recognizing and managing atypical coagulopathies.

The pathophysiology in our case can be conceptualized as a “triple-hit” model. First, IABP insertion as an intravascular foreign body, promoting contact factor activation and direct endothelial injury (8–10). Second, advanced heart failure, leading to low cardiac output and tissue hypoperfusion, fulfilling the “stasis” component of Virchow's triad (11). Third, catheter-related infection with systemic inflammation, amplifying coagulopathy via inflammatory cytokine-mediated activation of coagulation and impairment of natural anticoagulant pathways (12, 13). While these mechanisms are well-supported by indirect evidence, our literature search did not identify any prior case series or clinical studies describing IABP as an independent trigger for secondary hyperfibrinolysis. Notably, Onorati et al. (14) found that IABP-induced pulsatile flow during cardiac surgery ameliorates, rather than exacerbates, fibrinolytic activation. Thus, our case highlights a rare and potentially under-recognized complication, and suggests that clinicians should remain vigilant for the possibility of secondary hyperfibrinolysis in patients with multiple risk factors receiving IABP support.

A key diagnostic challenge was distinguishing isolated hyperfibrinolysis from classic disseminated intravascular coagulation (DIC). Routine coagulation tests (PT, APTT) were nonspecific, while dynamic monitoring of molecular markers was pivotal. Marked D-dimer elevation indicated intense fibrinolysis but was not specific for the underlying process (15, 16). The concurrent rise in PIC and TAT provided direct evidence of secondary hyperfibrinolysis associated with thrombin generation (17, 18). A stable platelet count and the absence of hypofibrinogenemia further excluded overt DIC or heparin-induced thrombocytopenia (19, 20). This case suggests that in IABP-supported patients, a marked D-dimer increase—especially in the context of infection or hemodynamic instability—should prompt consideration of atypical hyperfibrinolysis and trigger targeted molecular testing.

The use of TXA in this setting exemplifies pathophysiology-driven therapy. TXA competitively inhibits plasminogen activation, directly attenuating the hyperfibrinolytic process (21). While antifibrinolytic therapy in anticoagulated, IABP-supported patients carries theoretical thrombotic risk, careful risk-benefit assessment was crucial. Given active bleeding, evidence of marked hyperfibrinolysis (elevated PIC), and only mildly increased TAT, the benefits of TXA outweighed potential risks. The rapid decline in PIC and D-dimer after TXA confirmed efficacy, and no thrombotic complications occurred. Escalation of anticoagulation or empirical clotting factor replacement would have been inappropriate and potentially harmful in this context. Successful management required prompt laboratory diagnosis, multidisciplinary collaboration, and timely, targeted intervention.

This report is limited by its single-case nature, restricting generalizability. Optimal dosing and duration of TXA in this setting warrant further investigation. Additionally, comprehensive fibrinolytic profiling was not performed due to resource constraints. Finally, clinical decisions were guided by dynamic laboratory data and multidisciplinary clinical judgment, introducing an element of subjectivity.

## Conclusions

This case illustrates the marked instability of the coagulation system in end-stage heart failure, particularly during mechanical circulatory support. The interplay of IABP, infection, and advanced cardiac dysfunction can readily disrupt the balance between coagulation and fibrinolysis. Perioperative management should incorporate dynamic monitoring of fibrinolytic markers (such as D-dimer and PIC), alongside standard anticoagulation and infection control. When secondary hyperfibrinolysis with bleeding is identified, short-term TXA therapy under close monitoring may serve as a critical bridge to transplantation, ensuring patient safety during this high-risk period.

## Data Availability

The original contributions presented in the study are included in the article/Supplementary Material, further inquiries can be directed to the corresponding author.
